# A Monte Carlo Emissivity Model for Wind-Roughened Sea Surface

**DOI:** 10.3390/s19092166

**Published:** 2019-05-10

**Authors:** Jie Cheng, Xiaolong Cheng, Xiangchen Meng, Guanhua Zhou

**Affiliations:** 1 State Key Laboratory of Remote Sensing Science, Jointly Sponsored by Beijing Normal University and Institute of Remote Sensing and Digital Earth of Chinese Academy of Sciences, Beijing 100875, China; Jie_Cheng@bnu.edu.cn (J.C.); longtl93111@126.com (X.C.); xiangchenmeng@yeah.net (X.M.); 2 Beijing Engineering Research Center for Global Land Remote Sensing Products, Institute of Remote Sensing Science and Engineering, Faculty of Geographical Science, Beijing Normal University, Beijing 100875, China; 3 School of Instrument Science and Opto-Electronics Engineering, Beihang University, Beijing 100191, China

**Keywords:** sea surface emissivity, Monte Carlo model, ray-tracing, broadband emissivity, thermal-infrared, SST

## Abstract

Sea surface emissivity (SSE) is a key variable in the estimation of sea surface temperature and the sea surface radiation budget. A physical base SSE model with adequate accuracy and acceptable computational efficiency is highly desired. This paper develops a Monte Carlo ray-tracing model to compute the SSE of a wind-roughened sea surface. The adoption of a two-dimensional continuous surface model and averaging the two polarization components in advance before ray-tracing gives the model acceptable computational efficiency. The developed model can output the contributions of direct emission and the reflected component to the effective emissivity. The contribution of the reflected component to the effective emissivity reaches 0.035 at an 80° emission angle for a wind speed larger than 10 m/s. The emissivity spectra and channel emissivities collected from two field campaigns and one set of outdoor measurements are used to validate the developed model. Statistical results indicate that the absolute value of bias or difference is less than 0.5% when the view angle is less than 65°, which means the retrieval accuracy of sea surface temperature (SST) is guaranteed from the view of SSE. When the view angle increases, the accuracy of the developed model degraded, especially at the view angle of 85°. Without considering this view angle, the absolute value of bias or difference is less than 0.016, and the root mean square difference (RMSD) is less than 0.018.

## 1. Introduction

Sea surface emissivity (SSE) is the efficiency with which the sea emits its stored heat as thermal infrared radiation and is also an indicator of sea state including temperature, salinity and composition. In addition, SSE is a key parameter for an accurate estimate of sea surface temperatures (SSTs) from satellite radiometric measurements, which is a prognostic and a diagnostic variable in numerical weather prediction and in global climate modeling [1,2]. SSE and SST jointly determine the thermal radiation of the sea surface. The Earth’s energy imbalance (EEI) drives global warming [3,4], which can be best estimated from changes in the ocean heat content (OHC), complemented by radiation measurements from space [5]. However, the spectral variation as well as the directionality of SSE are ignored in calculating the sea surface energy balance, which will certainly affect the accuracy of the estimate of the sea surface radiation budget [6,7,8]. Additionally, current operational SST products cannot meet the desired goal of 0.3 K [2,9]. To achieve this goal, the accuracy of SSE is required to be 0.5% in the 8–12 µm spectral range. One of the primary causes for this relative lower accuracy of SST is that the SSE lacks accuracy [10,11]. It is highly urgent to improve the accuracy of SSE modeling or retrieval. 

So far, there are many studies on modeling the SSE of wind-roughened sea surfaces. The characteristic of sea surface geometry is modulated by the wind speed. First, a sea surface model is used to describe the geometry of the sea surface. For example, Cox and Munk proposed an isotropic slope distribution function with respect to the surface wind speed to describe the geometry of the sea surface [12]. Then, a physical or analytical model is constructed to characterize the interactions between the sea surface and photons. For example, Masuda et al. developed an analytical method to calculate the SSE [13]. They tabulated the SSE in the ranges of 3.5–4.1 μm and 8–13 μm for 11 view angles ranging from 0° to 85°. However, this model ignores the shadowing effects and multiple reflectances between wave facets, which inevitably introduces large deviations between the modeled and measured SSE. A difference of 0.03 between the model estimates and in situ measurements has been reported at larger emission angles. Watts et al. investigated the emission and reflection properties of rough sea surfaces and the role of surface-emitted surface-reflected (SESR) radiation, which enhances the emissivity value at high wind speeds [14]. Wu and Smith improved the model of Masuda et al. by incorporating the process of multiple reflections between wave facets [10]. They adopted a fixed piecewise function to include the effect of multiple reflections. According to a limited set of field measurements, the model of Watts et al. and the model of the Wu and Smith both enhanced the accuracy to a certain degree. Then, on the basis of the previous work, Masuda et al. improved their earlier model by accounting for SESR radiation [15]. They used a multi-order method to simulate the SSE. The first order is the same as the results of their earlier model, and the high order is performed by iterations. The high order emissivity means that there is one more iteration if there is one more reflection. In fact, the high order emissivity is a correction component of SESR. The discrepancy between the computed and measured emissivities at larger emission angles was significantly reduced to approximately 0.01 by incorporating the SESR emission compared to their earlier model. In addition, the isotropic Cox–Munk wave slope distribution function is used in the above models. Freund et al. developed the model using anisotropic Cox–Munk wave slope statistics [16]. Henderson et al. developed a model for calculating the polarized emissivity of wind-roughened sea surfaces using a Monte Carlo ray-tracing method [17]. They described the sea surface as a collection of interlocking isosceles triangles. Through a strict vector calculation, they computed the results accounting for the effects of both shadowing and the reflected component of the surface emission using the Monte Carlo method. Nalli et al. developed the surface-leaving radiance model to calculate the surface-leaving infrared radiance [18]. They allowed for atmospheric reflection into the model in a practical way. The model reduced the bias over standard models at emission angles larger than 45°, according to the validation [19]. 

Big progress has been made in modeling SSE. Some of the models described above have been used in the satellite remote sensing community. For example, the model of Wu and Smith is currently used within the Global Data Assimilation System of the National Centers for Environmental Prediction, U.S. National Oceanic and Atmospheric Administration [20]. Although some models are already used in real applications, large discrepancies between modeled and measured SSE still exist at greater observation angles. The accuracy of SSE modeling at large view angle is quite important. First, the local view angle may exceed 60° considering the large scan angle of the polar orbit satellite and local topography. Second, the calculation of hemispherical emissivity needs the directional emissivity at large view angle. The accuracy of SST retrieval and hemispherical emissivity calculation are guaranteed with the accurate directional emissivity at large view angle.

The purpose of this study is to develop a Monte Carlo ray-tracing model with acceptable computational efficiency to accurately compute the emissivity of wind-roughened sea surfaces. The adoption of a two-dimensional continuous surface model and averaging of the two polarization components in advance before ray-tracing gives the model acceptable computational efficiency. The use of a ray-tracing method allows us to include both the reflected emission and shadowing effects, which can guarantee the accuracy of the model. The remainder of the paper is organized as follows: the model description is introduced in Section 2. The results and validation are presented in Section 3. Section 4 discusses the main characteristics of the developed model, the estimate of broadband emissivity and limitations of this study, and Section 5 is the conclusion.

## 2. Model Description

### 2.1. Sea Surface Model

**Figure 1:**
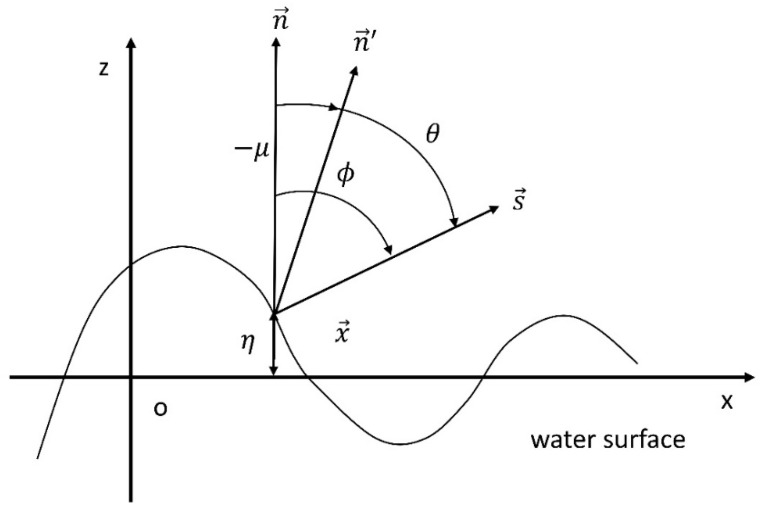
Cartesian coordinate system. (Modified from Yoshimori et al. [21]).

We adopt a simple surface model to describe the sea surface [21]. As shown in Figure 1, the x and y axes in the Cartesian coordinate system are on the average water surface, whose unit normal $\vec{n}$ is parallel to the z axis. The surface displacement at the point on the average surface is specified by $\left(\vec{x},0,0\right)$ and is denoted as $\eta (\vec{x},t)$ at time t. For our study of the optical characteristics, we address the two-dimensional shape of the random water surface, the cross-section that the surface makes with the x−z plane. This means that the y component of the surface slope is omitted. This approximation of no y component is valid for a small surface slope [22]. The observation of the surface is made from the direction specified by the unit observation vector:

(1) $$\vec{s}=(\sin\phi ,0,\cos\phi ),$$

where ϕ denotes the viewing angle. We introduce a unit normal ${\vec{n}}^{\prime }$ to the local surface surrounding a point specified by the position vector $x=[\vec{x},0,\eta (\vec{x},t)]$ and let the angle between $\vec{s}$ and ${\vec{n}}^{\prime }$ be θ; that is,

(2) $${\vec{n}}^{\prime }\cdot \vec{s}=\cos\theta .$$

Since it is assumed that ${\vec{n}}^{\prime }$ has no y component, the surface slope is defined by:

(3) $$\gamma =\frac{\partial \eta (x,t)}{\partial x},$$

where μ denotes the local angle of the surface slope. It is clear that θ is given by:

(4) θ=ϕ+μ.

After establishing the water surface model, we need to specify the statistical characteristics of the water surface. It has been experimentally confirmed that the surface displacement of the ocean surface obeys the normal distribution with a slight asymmetry [23], and the distribution of the surface slope can be approximated as a Gaussian random process,

(5) $$P(\gamma )=\frac{1}{\sqrt{2\pi }\gamma_{0}}\exp(-\frac{\gamma^{2}}{2\gamma_{0}^{2}}),$$

where $\gamma_{0}$ denotes the root mean square of the surface slope. To simulate a wind-roughened sea surface, we refer to the facet slope distribution function of the wind-roughened sea surface proposed by Cox and Munk [12]. The root mean square of the surface slope is expressed as a function of wind speed:

(6) $$2\gamma_{0}^{2}=0.003+0.00512w,$$

where w is the wind speed at masthead, i.e., 12.5 m above the sea surface.

### 2.2. Ray Tracing

Monte Carlo approach provides the most general and flexible technique for radiative transfer calculation [24]. It has been used in the ocean optics community over the past decades. Monte Carlo simulation can provide new information, insight or understanding and infer conclusions from a sequence of stochastic process. The way to model the SSE using Monte Carlo method is to follow the particles of interest one at a time through all their interactions. The ‘forward’ Monte Carlo technique requires a tremendous number of rays since the wind-roughened water surface is a complex multi-scale surface with essentially an infinite number of possible ray path geometries leaving the surface. The ‘reverse’ or ‘backward’ technique was used in this study to compute the emitted ray paths. We traced photons from the detector to the source rather than from the source to the detector. The reverse Monte Carlo techniques allow us to only follow photons that are pertinent to the final outcome of the simulation which will improve the computation efficiency. 

**Figure 2:**
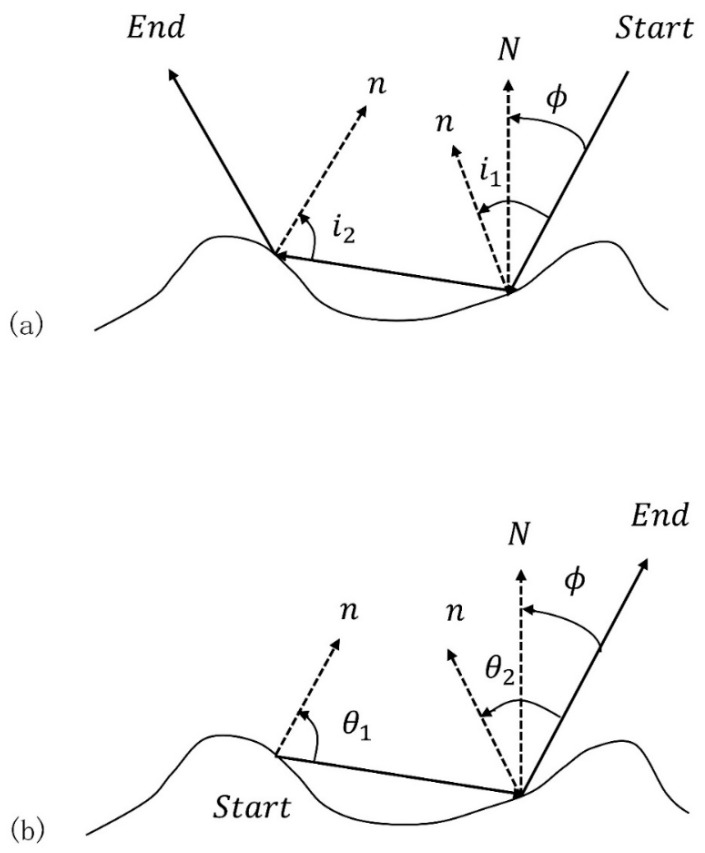
Sketch showing the reverse approach used to calculate the geometry of an individual ray path for emitted photons exiting the surface at an angle ϕ relative to the average surface. In (**a**), the motion is downward from the ‘Start’ along a ray path defined by the angle ϕ. Specular reflection is assumed when the ray strikes another facet on the surface. Motion ceases when the ray leaves the surface or reaches a predetermined maximum number of reflections. The emission is computed by reversing the direction of the ray path, using the point of the last reflection (‘Start’ in (**b**)) as the initial source of radiation along the ray. (Modified from Henderson et al. [17]).

As shown in Figure 2, to implement the reverse Monte Carlo simulation, we start at a point above the surface and move downward toward the central facet along a ray path predefined by the angle ϕ that the ray makes with the normal N to the average surface (the angle ϕ will eventually be the emission angle for the ray path, see Figure 2a). When the ray strikes a facet, its local angle of incidence I is recorded, and its subsequent trajectory is defined by specular reflection. We repeat this procedure facet by facet until the ray bounces back to the sky or reaches a set maximum number of reflections. We then calculate the intensity of the radiation emitted along that same ray path by traversing it in the opposite direction. We go to the point of the last reflection and use that facet as the initial source of radiation along the ray (Figure 2b). The basic idea is that if an imaginary ray can follow that path going toward the surface, then actual emitted photons can escape along the same path. The advantage of this reverse approach is that it lets us specify a desired viewing geometry at the start of a run. At the source facet, the normal n and the exiting ray define the plane of emission. The angle between the normal and the ray is the local emission angle θ. Since the facet is a smooth surface, it will be a Fresnel emitter with emissivity ε(θ;λ) determined by the angle θ and the complex refractive index n(λ) (a function of wavelength λ). This value is computed via Kirchhoff’s Law,

(7) ε(θ;λ)=1−ρ(θ;λ),

where ρ(θ;λ) is the Fresnel power reflectivity:

(8) $$\rho (\theta ;\lambda )=\frac{1}{2}\left\{{\left[\frac{\tan(\theta -\theta^{\prime })}{\tan(\theta +\theta^{\prime })}\right]}^{2}+{\left[\frac{\sin(\theta -\theta^{\prime })}{\sin(\theta +\theta^{\prime })}\right]}^{2}\right\},$$

(9) $$\sin\theta^{\prime }=\frac{1}{n(\lambda )}\sin(\theta ).$$

**Figure 3:**
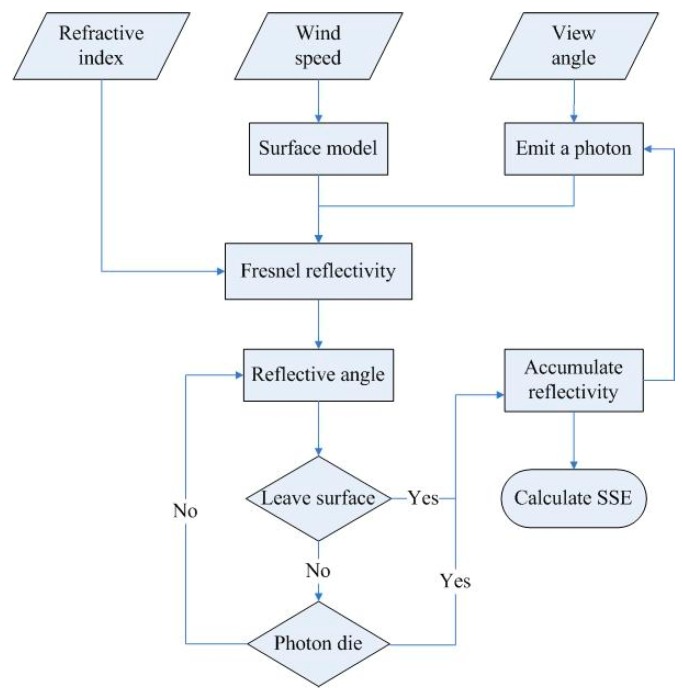
Flowchart describing the procedure for calculating the sea surface emissivity (SSE).

To begin a model run, the wind speed in meters per second at 12.5 m above the sea surface, view angle and complex refraction index are required. The flow chart is shown in Figure 3. Following are the detailed steps:

(1) Generate the sea surface model randomly using the provided wind speed.
(2) Select a certain point randomly above the sea surface as the starting point. Then, emit a photon along the given view angle, and the photon strikes the sea surface. Assume that the initial energy weight is one.
(3) Calculate the crossover point between photon and sea surface. Assume that each collision is specular reflection and then compute the incident angle and reflection angle.
(4) Following the law of Fresnel and Snell, a single-pass reflectivity is computed by using the complex refraction index as a parameter.
(5) After each collision, the energy weight of a photon multiplies by single-pass reflectivity and we get a new weight of energy.
(6) Then, the reflected photon keeps hitting the surface. If the photon leaves the surface, the process stops and calculate the energy of reflection. If not, repeat step (3).
(7) Continue the simulation process until the energy of photon is less than a certain value or the number of bounces reaches the maximum.
(8) In the Monte Carlo method, we compute huge numbers of photons and repeat step (1) to step (7) until the result is close enough to the real value.
(9) Finally, calculate the average of the reflected energy, and subtract it from one to get the effective emissivity according to Kirchhoff’s law.

Finally, we then get the emissivity at a certain view angle. We can get the directional emissivity at other wavelengths using the same method. Additionally, we can calculate the channel emissivity and broadband emissivity using the simulated directional emissivity spectra, and the expressions are given as follows:

(10) $$\varepsilon_{ch}(\mu )=\frac{\int_{\lambda_{1}}^{\lambda_{2}}\varepsilon (\mu )f(\lambda )d\lambda }{\int_{\lambda_{1}}^{\lambda_{2}}f(\lambda )d\lambda },$$

(11) $$\varepsilon_{bb}(\mu )=\frac{\int_{4}^{100}\varepsilon (\mu )B_{\lambda }({Τ}_{s})d\lambda }{\int_{4}^{100}B_{\lambda }({Τ}_{s})d\lambda },$$

where $\varepsilon_{ch}(\mu )$ is the channel emissivity at observation angle θ, $\lambda_{1}$ and $\lambda_{2}$ are the lower and upper bounds of wavelength integration, respectively, and f(λ) is the instrumental filter function. $\varepsilon_{bb}(\mu )$ is the directional broadband emissivity at view angle μ, and $B_{\lambda }({Τ}_{s})$ is the Planck function at a surface temperature of ${Τ}_{s}$, which is often set as 300 K. The hemispherical broadband emissivity can be calculated by the angular integration of $\varepsilon_{bb}(\mu )$.

## 3. Results and Validation

### 3.1. Model Results

**Figure 4:**
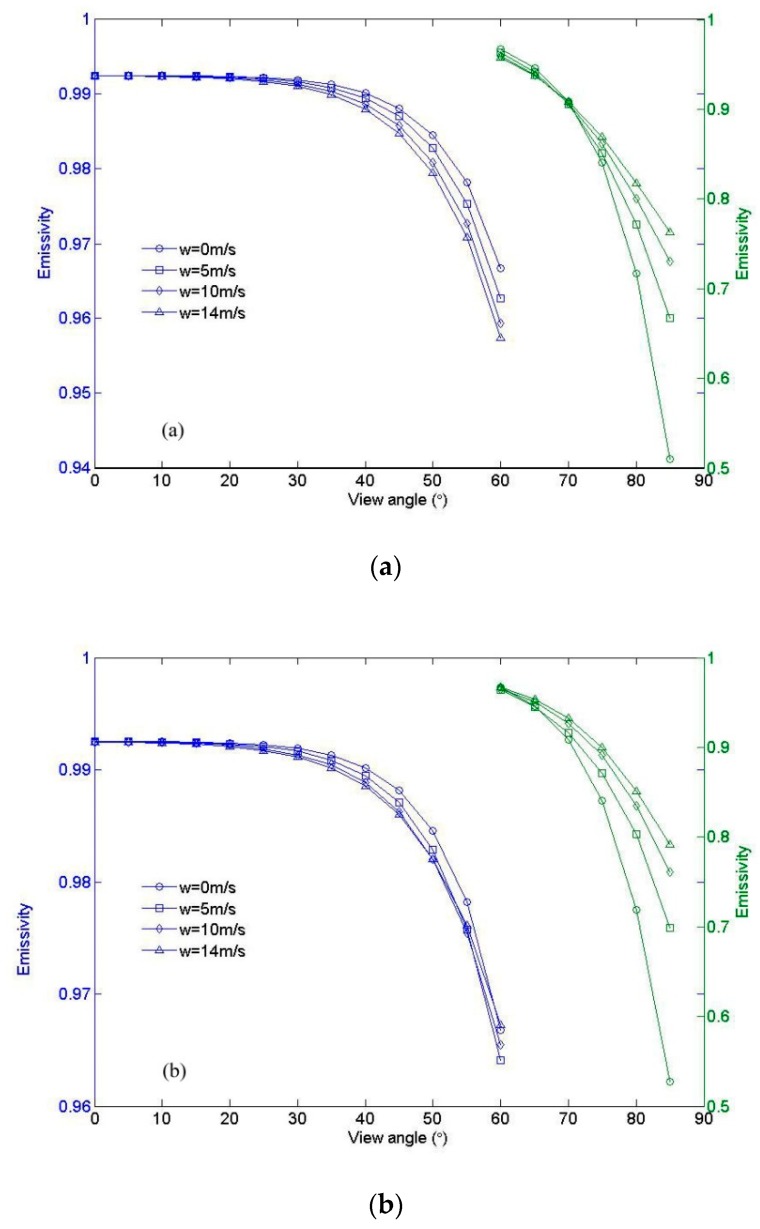
The results in (**a**) were calculated with a single interaction and do not include the reflected component, only direct emission from surface roughness; in (**b**), there are no limits and therefore roughness, shadowing, and reflections are all included. In (a) and (b), the left y axis applies to 0°–60°, whereas the right y axis applies to angles larger than 60°.

**Figure 5:**
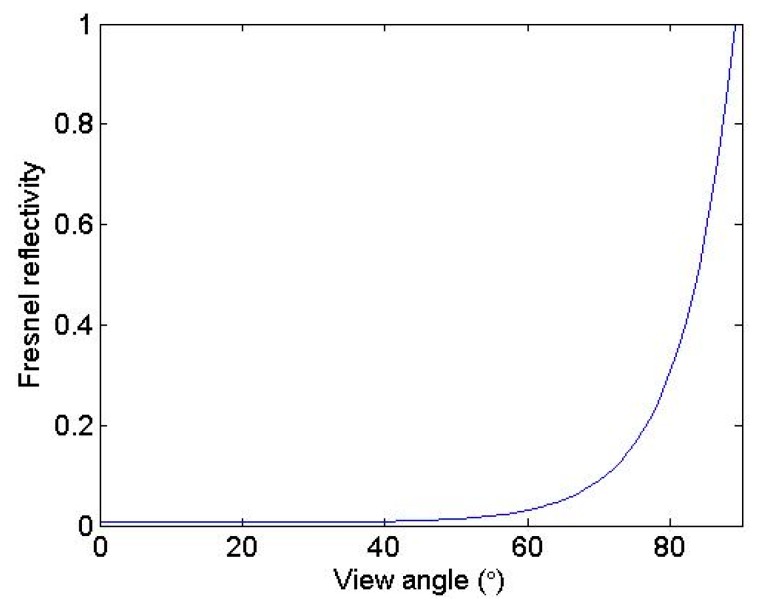
The Fresnel reflectivity of sea water at a wavelength of 11 µm.

The refractive index of pure water by Hale and Querry [25] with the salinity adjustment from Friedman [26] is adopted in this study. The simulated directional emissivities of the sea water surface at 11 µm at emission angles of 0°–85° for wind speeds 0, 5, 10 and 14 m/s are shown in Figure 4. For clarity, the plots have been split so that the left y axis applies to angles less than 60°, and the right y axis applies to angles greater than 60°. Figure 4a shows the direction emissivity that was computed with just one reflection, which means that these curves represent only the direct component of the emission without considering the effect of reflections. In this situation, the variation in emissivity with respect to wind speed is due to changes in the observed slope distribution, weighted by the Fresnel emissivity, integrated over all the facets that are visible at the given viewing geometry. Theoretically, the emissivity in Figure 4a should be equivalent to that of Masuda et al. [13]. Note that for angles less than approximately 60°, increased surface roughness due to increased wind speed reduces the effective emissivity. In this situation, the surface approximates a Fresnel emitter. The Fresnel reflectivity is shown in Figure 5. The Fresnel curve decreases monotonically with the increasing emission angle and reaches a maximum at 0°. Thus, integrating the Fresnel curve weighted wave-slope probability distribution brings in enough high angles for angles less than approximately 60°. Low-emissivity components reduce the effective emissivity below what it would be for a smooth surface. At higher angles, the situation is reversed, with a crossover point occurring between 60° and 70°, and increasing surface roughness tends to increase the effective emissivity above this angle. The crossover point is not constant in different situations, but it is always in the range between 60° and 70°. 

The model results that include the effects of roughness, shadowing, and multiple reflections are shown in Figure 4b. Note that at low ($\phi \leq 45^{\circ }$) and high ($\phi \leq 70^{\circ }$) emission angles, the plots are qualitatively the same as before. The effective emissivity decreases at low angles and increases at high angles with increasing surface roughness. However, the behavior in which the wind speed increases the effective emissivity at intermediate angles is quite different from that at high angle. This effect is due to the contribution of the reflected component of the emitted field. As the fraction of reflected rays increases, the effective emissivity increases. As the contribution of reflections increases with surface roughness, the effect is more pronounced at high wind speed.

**Figure 6:**
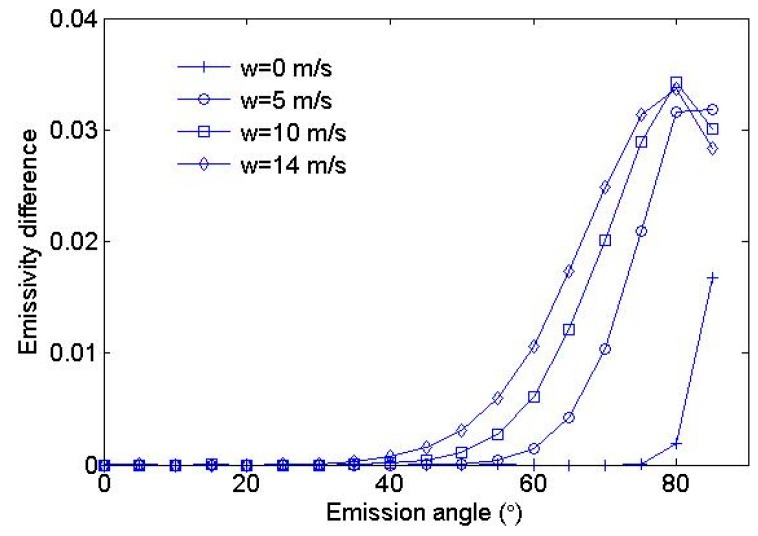
The fraction of reflected ray paths as a function of emission angle at four wind speeds. These curves quantify the fraction of the emitted ray paths that reflected off of one or more surface facets after being emitted by the source facet.

Figure 6 shows the fraction of the reflected component as a function of emission angle. The maximum of the reflected component is approximately 0.035 when the emission angle is approximately 80° and the wind speed lies between 10 and 15 m/s. In this case, we also recorded the reflection times of each photon. At a view angle of 60°, there are nearly 11,176 reflections per 100,000 photons when the wind speed is 15 m/s. In addition, we find that the number of reflections of most photons are within five through the studying of a single photon, and there are hardly any photons whose number of reflections is larger than 5. It is easy to understand this phenomenon. As shown in Figure 5, the Fresnel reflectivity at 60° is less than 0.1. If we assume an extreme situation in which every reflection has this largest reflectivity, the 5th power of 0.1 is 1.0 × 10^5^. This value is so small that we can consider that the photon dies during the multiple reflection process. Among the photons that have reflections, most of them have two or three reflections. In total, approximately 10% of photons participate in the process of the multiple reflection process. The reflected component is thus a significant contributor to the effective emissivity.

### 3.2. Validation

#### 3.2.1. Field Measurements

Field measured SSEs are rarely scarce. Two widely used sea surface SSE emissivity data and one newly published SSE emissivity data were used to validate the developed model in this study, including (1) the emissivity spectra from Smith et al. were derived from the measurements collected by a high spectral resolution Fourier Transform Spectrometer (FTS) aboard an oceanographic research vessel at three view zenith (36.5°, 56.5°, 73.5°) with the accuracy of 0.1% [27]; (2) Niclòs et al. [28] measured the SSE in the Mediterranean Sea by using a Cimel Electronique CE 312 radiometer with four channels (Ch1: 8–14 μm; Ch2: 8.2–9.2 μm; Ch3: 10.5–11.5 μm; Ch4: 11.5–12.5 μm). The multi-angle SSE were collected with the observation angles ranging from 25° to 65° with a step of 5° under wind speeds of approximately 5 and 10 m/s, respectively. The accuracy of the derived sea surface emissivity was claimed to be ±0.004. (3) Branch et al. [29] measured the emissivity spectra of the generated foam at the band of 3.5–5.5 μm and 8–14 μm with large observation angles ranging from 60° to 85°. The corresponding directional emissivity spectra of foam-free sea water were also measured. For detailed information on the measurements and the technique for deriving the corresponding emissivity data, please refer to their respective papers [27,28,29]. 

#### 3.2.2. Validation Results

With the wind speeds and view angles the same as those during the field measurements, we simulated the emissivity spectra of the sea surface using the developed Monte Carlo ray-tracing model. The simulated emissivity spectra were either compared to the measured emissivity spectra directly or integrated with the instrumental filter functions to produce channel emissivity or broadband emissivity and compared to the measured channel emissivity or broadband emissivity next. 

**Figure 7:**
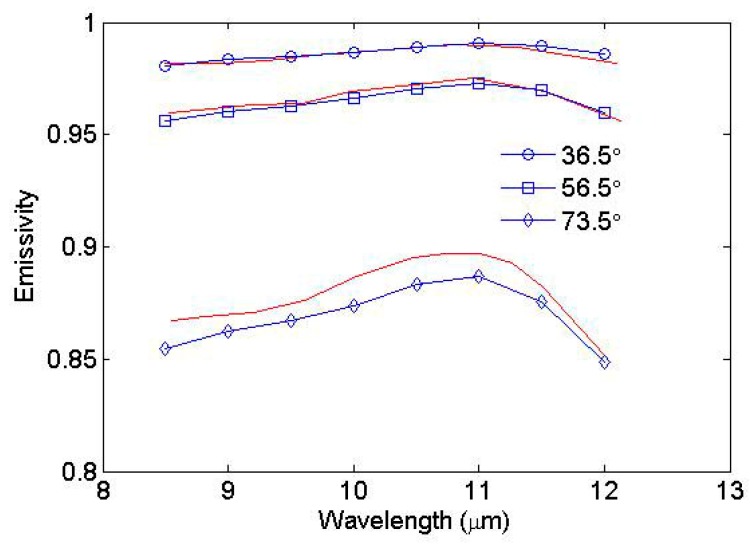
Simulated directional emissivity spectra versus in situ measured emissivities for view angles of 36.5°, 56.5° and 73.5°. The measured emissivities (red lines) are reproduced from Figure 10 by Smith et al. [27].

Figure 7 shows the comparison results between the simulated sea surface emissivity spectra and those measured by Smith et al. at 8–12 μm [27]. The wind speed is set as 5 m/s in the simulation, because the wind speed was approximately 5 m/s on the day that the emissivity spectra were measured. The modeled sea surface emissivity spectra agree nicely with the measured values when the view angles are 36.5° and 56.5°. When the view angle is 73.5°, the discrepancy is pronounced. The maximum difference is −0.013. The statistical results are shown in Table 1. The bias and RMSD are −0.009 and 0.0102 respectively at a view angle of 73.5°, and the bias is less than 0.001 and the RMSD is less than 0.0021 at other view angles. This accuracy is comparable to that of Masuda [15] and Henderson et al. [17]. 

**Figure 8:**
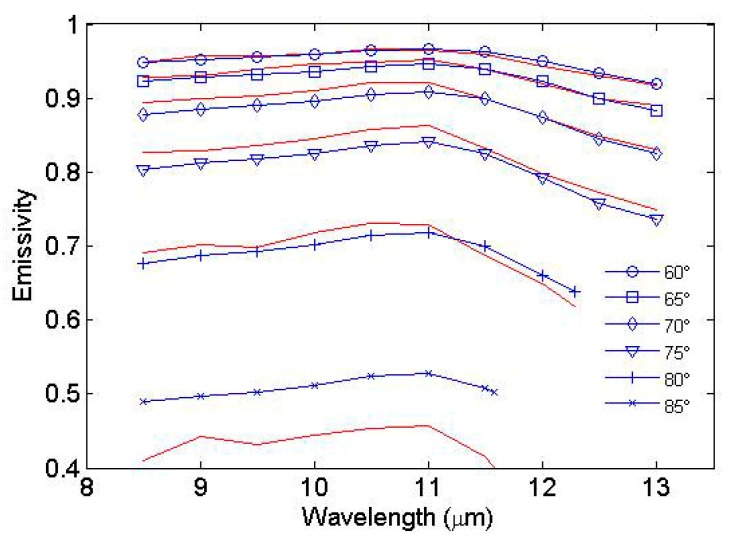
Comparison between simulated emissivity spectra and those measured by Branch et al. [29] at larger view angles. The measured emissivities (red lines) are reproduced from Figure 9 by Branch et al. [29].

Figure 8 shows the comparison between the simulated angular sea surface emissivity spectra and the emissivity spectra measured by Branch et al. [29]. The statistical performance of the developed model is provided in Table 2. When the view angle is less than 65°, the simulated emissivity spectra show good agreement with the measurements. The absolute value of bias is less than 0.004, and the RMSD is less than 0.006. The agreement degrades with the increasing view angle. The absolute value of bias and RMSD are less than 0.016 and 0.0181, respectively, when the view angles are 70°, 75° and 80°. The emissivity is obviously overestimated when the view angle is 85°, with the bias and RMSD of 0.0757 and 0.0822, respectively.

Regarding the validation using the measurements of Niclòs et al. [28], we first simulated the directional emissivity spectra under wind speeds of 4.5 and 10.3 m/s, then calculated the channel emissivity by convoluting with the channel filter functions using Equation (10). The comparisons are shown in Table 3. The differences for the two wind speeds are not significant when the view angle is less than 55°. The absolute differences are less than 0.0025, 0.0025, 0.0008 and 0.004 for channels 1, 4, 3 and 2, respectively. These differences are lower than the uncertainty in the measured SSE values, which is approximately 0.004 [28]. When the view angle equals 65°, the absolute differences become larger, ranging from 0.0009 to 0.0111. 

Combining the results provided in Table 1, Table 2 and Table 3, the differences under most conditions are less than 0.5%, which means the retrieval accuracy of SST is guaranteed from the view of SSE. When the view angle is larger than 65°, the accuracy of the developed Monte Carlo model degraded, especially at the view angle of 85°. Without considering this special view angle, the absolute value of bias or difference is less than 0.016, and the RMSD is less than 0.018. 

## 4. Discussion

### 4.1. Features of the Newly Developed Model

In brief, the developed Monte Carlo ray tracing model has two features: (1) adopt a continuous curve to describe the sea surface. Constructing a suitable sea surface model is important in the development of the Monte Carlo ray-tracing model. We adopted a continuous curve to describe the sea surface, which strictly complies with the statistical model of Cox and Munk [12]. (2) Improve the computational efficiency of ray tracing. Ray tracing is generally time consuming. Our ray tracing model averages the two polarization components in advance and performs the vector calculations in the following step. Combined with the continuous surface model, the calculation steps are simplified, and computational time is saved.

### 4.2. Potential Use of the Model

Another application of the SSE simulated by the model is to estimate sea surface hemispherical broadband emissivity, which is the angular integration of directional hemispherical broadband emissivity (Equation (11)). The hemispherical broadband emissivity is an important parameter for calculating the sea surface energy balance. Cheng et al. [6] developed a lookup-table based algorithm for estimating hemispherical broadband emissivity using the SSE model of Wu and Smith [10]. Even the Wu and Smith model cannot reproduce well the observations of Smith et al. [27] and Branch et al. [29]. At a larger view angle, the error on the estimate of hemispheric broadband emissivity is greatly reduced through angular integration, and the accuracy is 0.003 under wind free conditions. The accuracy of the developed model in this study is comparable to the accuracy of the model of Wu and Smith [10], so we can obtain highly accurate hemispherical broadband emissivity from the directional emissivity spectra simulated by the developed model.

### 4.3. Limitations of this Study

Even though the modeling accuracy of SSE has been improved continuously, there is still a lot of work to do. For example, an absolute accuracy of 0.3 K for satellite-retrieved SSTs is desired for applications in climate monitoring and operational oceanography. The emissivity needs to be determined with an accuracy of 0.5%. However, the absolute differences or mean absolute bias are larger than 0.01 when the view angle is larger than 65° according to the validation results in this study. The SSE model may be improved through four steps: 

(1) Improve the accuracy of the sea water complex refractive index

The complex refractive index is one of the key inputs of the SSE model. Different authors have published various complex refractive indices for pure water and sea water [25,30]. Larger discrepancies occur in these indices [6]. More laboratory or field measurements should be deployed to obtain high quality data of the complex refractive index. For the practical application, the global classification of sea water complex refractive index is an option since the refractive index varies with the salinity. 

(2) Construct a unified water surface model

Different water surface models, such as the model of Cox and Munk [12], Preisendorfer and Mobley [31], and the continuous curve model [21], are used to describe the water surface. To study the model mechanism and to make the modeling results comparable, a unified and accurate water surface model is desired. 

(3) Improve the accuracy of SSE measurements

Field emissivity measurements using FTS is not an easy task, especially the SSE [32]. First, the temperature of the detector of FTS should be stable and avoid noise and background disturbance [32]. Then, an accurate temperature and emissivity separation algorithm is required [33]. According to the field measurements of land surface emissivity using FTS, an accuracy of 0.01 can be achieved [34]. The environment is more complex at the sea surface than that at the land surface. The measurement accuracy of the same instrument may be degraded at the sea surface compared to the land surface due to environmental disturbance. 

(4) Incorporating the effects of whitecaps

When the wind speed is larger than 10 m/s, significant whitecaps or foam appear on the sea surface. The optical characteristics of whitecaps are quite different from sea water [35]. The real SSE should be the weighted average of SSE and whitecap emissivity. Currently, the whitecap coverage product and laboratory measured emissivity of whitecaps or foam are available [35,36]. The effect of foam is incorporated in the retrieval sea surface broadband emissivity empirically [6]. More studies should be conducted to study the emissivity and coverage of whitecaps. The effects of whitecaps should be incorporated into sea surface models physically.

## 5. Conclusions

We developed a Monte Carlo ray-tracing model to simulate the emissivity of a wind-roughened sea surface. The adoption of a two-dimensional continuous surface model and averaging the two polarization components in advance before ray-tracing gives the model an acceptable computation efficiency. The use of a ray-tracing method allows us to include both the reflected emission and shadowing effects, which guarantees the accuracy of the model. The developed model can output the contributions of direct emission and the reflected component. There is a special angle when the reflected component begins to appear. The special angle decreases when the wind speed increases. The maximum of the reflected component is approximately 0.035 at an 80° emission angle for a wind speed larger than 10 m/s. The emissivity spectra and channel emissivities collected from two field campaigns and one set of outdoor measurements are used to validate the developed model. Generally, the simulations agree well with the measurements. The accuracy of the developed model is comparable to the model of Masuda [15] and Henderson et al. [17] when validating by the field measurements of Smith et al. [27]. Statistical results indicate that the absolute value of the bias or difference is less than 0.5% when the view angle is less than 65°, which means the retrieval accuracy of SST is guaranteed from the view of SSE. When the view angle increases, the accuracy of the developed Monte Carlo model degraded, especially at the view angle of 85°. Without considering this view angle, the absolute value of bias or difference is less than 0.016, and the RMSD is less than 0.018. The degraded simulation results at a larger view angle will not affect the accuracy of the estimate of hemispherical broadband emissivity. 

## Figures and Tables

**Table 1:** The bias and root mean square difference (RMSD) values between the simulated and measured sea surface emissivity values.

View Angle	36.5°	56.5°	73.5°
Bias	0.001	−0.0014	−0.009
RMSD	0.0017	0.0021	0.0102

**Table 2:** The bias and RMSD values between the simulated sea surface emissivity values and the measurements of Branch et al. [29].

View Angle	60°	65°	70°	75°	80°	85°
Bias	0.0008	−0.004	−0.010	−0.0160	−0.0038	0.0757
RMSD	0.0036	0.006	0.0128	0.0181	0.0150	0.0822

**Table 3:** The difference between simulated and observed channel emissivities.

View Angle	Channel 1 Diff.		Channel 4 Diff.		Channel 3 Diff.		Channel 2 Diff.	
	ws1	ws2	ws1	ws2	ws1	ws2	ws1	ws2
25°	−0.0006	−0.0009	−0.0022	−0.0025	0.0001	−0.0002	0.0027	0.0023
35°	−0.0005	−0.0013	−0.002	−0.0016	0.0008	−0.0007	0.0028	0.002
45°	−0.0003	−0.0007	−0.0002	−0.0005	0.0007	0.0007	0.004	0.0005
55°	−0.0025	−0.0023	0.0019	0.0012	0.0008	0.0002	0.0025	0.0004
65°	−0.0111	−0.0056	−0.0019	0.0053	−0.0027	0.0009	−0.0057	−0.0013

Note: ws1 = 4.5 m/s, ws2 = 10.3 m/s. Channel difference equals to the modeled minus the observed channel emissivities.
